# The C57BL/6N mouse substrain is a viable model of elastase-induced abdominal aortic aneurysm

**DOI:** 10.3389/fcvm.2024.1462032

**Published:** 2024-09-27

**Authors:** Panpan Wei, Kexin Li, Meng Li, Haole Liu, Congcong Xia, Yajie Chen, Sihai Zhao, Yankui Li

**Affiliations:** ^1^ Department of Vascular Surgery, The Second Hospital of Tianjin Medical University, Tianjin, China; ^2^ Institute of Cardiovascular Science, Translational Medicine Institute, Xi'an Jiaotong University Health Science Center, Xi’an, Shaanxi, China; ^3^ Guangdong Provincial Key Laboratory of Large Animal Models for Biomedicine, School of Pharmacy and Food Engineering, Wuyi University, Jiangmen, Guangdong, China

**Keywords:** abdominal aortic aneurysm, smooth muscle cell, inflammation, elastin, animal model

## Abstract

**Aim:**

Compared with the C57BL/6N substrain, the C57BL/6J substrain is more susceptible to the angiotensin II (Ang II)-induced development of dissected abdominal aortic aneurysms (AAAs). The aim of this study was to elucidate whether the widely used C57BL/6N mouse substrain is as susceptible as the C57BL/6J mouse substrain to porcine pancreatic elastase (PPE) infusion-induced experimental nondissected AAA development.

**Methods:**

Experimental nondissected AAAs were induced in C57BL/6J and C57BL/6N mice via transient aortic luminal infusion of PPE. On Day 0 (baseline) and Day 14 after PPE infusion, the abdominal aortic diameter was directly measured. Aortic aneurysmal segment samples were collected, and histopathological analysis was performed.

**Results:**

On Day 14 after PPE infusion, aortic diameters were significantly increased in both mouse substrains (from approximately 0.51 to 1.24 mm in C57BL/6J mice and from 0.51 to 1.18 mm in C57BL/6N mice). The increase in diameter of all the mice exceeded 50% and met the criteria for AAA model establishment (143% and 135% in C57BL/6J mice and C57BL/6N mice, respectively). PPE infusion also induced obvious local aortic wall macrophage and *T*-cell infiltration, elastin degradation, smooth muscle cell depletion and high metallopeptidase (MMP)-2 and MMP-9 expression levels in C57BL/6N mice, but these differences were not significant compared with those in C57BL/6J mice. However, PPE infusion led to the recruitment of more B cells and the sprouting of more neovessels at the aneurysmal lesion site in C57BL/6J mice than in C57BL/6N mice.

**Conclusion:**

The C57BL/6N mouse substrain is suitable for establishing a model of AAA via elastase infusion.

## Introduction

Abdominal aortic aneurysm (AAA) is a major aortic disease characterized by local expansion of the abdominal aorta (1–3). Histopathologically, aortic aneurysmal lesions are characterized by depletion of medial smooth muscle cells (SMCs), breakage or disappearance of medial elastin, degradation of the extracellular matrix, and local inflammation in human and experimental animal models (2–6). The ability of the aneurysmal aorta to withstand the stress of the blood flow load is reduced, and rupture of the aneurysm is the main cause of death in most patients with AAAs (1, 2). To date, endovascular aneurysm repair (EVAR) is still the main clinical intervention for treating AAA, which also makes it difficult to collect clinical AAA samples in laboratory studies. Therefore, many AAA studies have been performed in experimental animal models.

Rodents are the most commonly used models to study the pathogenesis of AAA and treatments (3–5). Owing to the standardized production of mice, the large number of strains, and the maturity of gene editing technology, the advantages of mice in AAA research are becoming increasingly prominent. However, different strains or substrains of mice have different susceptibilities to the same modeling method when modeling cardiovascular diseases, including AAA (5, 7–9). The commonly used methods for establishing AAA models include transient porcine pancreatic elastase (PPE) pressured infusion, Ang II chronic infusion via osmotic pumps, and periaortic application of calcium chloride (CaCl_2_), and the first two methods are more commonly used (6, 9, 10). C57BL/6 is the most popular strain, and one of the C57BL/6 substrains, C57BL/6J, is reported to be more susceptible than the C57BL/6N substrain to the angiotensin II (Ang II)-induced development of dissected AAAs (9). It remains unclear whether the C57BL/6N mouse substrain is as susceptible as the C57BL/6J substrain to PPE infusion-induced nondissected AAA model establishment.

In this study, PPE infusion was used to induce nondissected AAAs in two widely used mouse strains, C57BL/6N and C57BL/6J, and to compare their sensitivity to PPE challenge. We found that the response of C57BL/6N and C57BL/6J mice to PPE infusion-induced AAA model establishment was similar, and no significant differences were found. The C57BL/6N mouse substrain could be used for nondissected AAA research.

## Materials and methods

### Establishment of experimental AAAs in mice

Nine-week-old male C57BL/6N or C57BL/6J mice were purchased from Beijing Vital-star Biotechnology Co., Ltd (Beijing, China). Experimental AAAs were induced in all the mice according to previously described methods (11–13). Type I PPE for infusion was freshly prepared in phosphate-buffered saline (PBS) (1.5 U/ml, Cat #, E-1250; Sigma–Aldrich, St. Louis, USA). After the mice were anesthetized with 2% isoflurane inhalation, a 2–3 cm long incision was made along the midline of the abdomen, the abdominal aorta was bluntly separated, and the branch aortas were ligated to avoid leakage during PPE infusion. After the infrarenal aorta was temporarily ligated, aortotomy was performed via a 30-gauge needle. A PE-10 infusion tube was inserted into the abdominal aorta, and PPE solution was pressure infused for 5 min with a continuous infusion pump. Afterward, the aortotomy was closed with 11-0 sutures, and then the abdominal cavity was closed with 4-0 sutures. After a few hours of postoperative recovery, the mice were returned to the feeding room and maintained for 14 days.

### Measurement of blood pressure

Blood pressure were also measured in two new separate cohorts of C57BL/6N and C57BL/6J mice when we revised this paper. The noninvasive blood pressure was measured by a BP-2000 system (a tail-cuff Method, Visitech Systems, Inc., Apex, NC). Briefly, the mouse was placed in a holder on the warming platform. Approximately ten measurements were taken with the machine for each animal, and the final mean value was calculated and recorded.

### Aortic diameter measurement and specimen collection

The diameter of the abdominal aorta was directly measured as previously described (4, 5). Briefly, prior to PPE infusion (baseline, recorded as Day 0) and 14 days after surgery (recorded as Day 14), the abdominal aortas of the mice were photographed under a stereomicroscope, and the aortic diameter was directly measured by image software (13). If the expansion of the aortic diameter on day 14 exceeded 50% of the basal diameter (day 0), the AAA model was considered to have been successfully established (6, 11). To measure the inner aortic diameters continuously by ultrasound machine (Vevo® LAZR-X, Fujifilm VisualSonics, Inc, Toronto, ON, Canada), we performed surgery for PPE intraluminal infusion in two new separate cohorts of C57BL/6N and C57BL/6J mice. Serial transabdominal ultrasonographic images were photographed to monitor the progression of experimental AAAs. Maximal internal infrarenal diameter of the infused aortic segment was documented by investigators. After 14 days of PPE infusion, the mice were euthanized by carbon dioxide overdose inhalation, the abdominal aorta of the dilated segment was removed, and the fresh aortic samples were embedded in optimal cutting temperature (O.C.T.) compound for subsequent sectioning. Frozen aortic sample blocks were used for serial sectioning (6 μm), followed by histopathological analysis.

### Histological analysis of experimental AAAs

Frozen sections were subjected to hematoxylin‒eosin (H&E) staining and elastic van Gieson (EVG) staining according to the manufacturer's instructions. Immunohistochemistry (IHC) was performed on serial frozen sections via the standard biotin‒streptavidin peroxidase method (6). For histological and IHC analysis, adjacent non-aneurysmal healthy abdominal aorta samples were used as negative control. The primary antibodies that were used in the IHC analysis included anti-smooth muscle alpha-actin antibodies to label vascular SMCs; antibodies that label infiltrated leucocyte subsets, including macrophages (anti-CD68), *T* cells (anti-CD4 and anti-CD8) and B cells (anti-CD45R); antibodies that detect the expression levels of matrix metallopeptidase (MMP)-2 and MMP9 in aneurysm lesions; and endothelial cell antibodies (anti-CD31) that mark abnormal mural angiogenesis. The detailed dilutions and source information for the primary antibodies, secondary antibodies and related reagents that were used for histological detection are summarized in Supplementary Table S1. In accordance with a previously reported method, the severity of medial elastin degradation/break was scored as I–IV from mild to severe (5, 6). The degrees of medial SMC depletion and macrophage infiltration were also semiquantitatively analyzed via histological grading on the basis of criteria from previous studies. *T* cells and B cells were quantified by counting the cell number per aortic cross section (ACS). MMP-2 and MMP-9 were analyzed by calculating the positive staining area per ACS via image software (WinRoof 6.5, Mitani Co. Ltd., Tokyo, Japan). Angiogenesis of aneurysmal lesions was analyzed by counting the number of neovessels in each cross section.

### Zymography of aneurysmal aortas

*in situ* zymography was conducted by using a gelatinase/collagenase assay kit (Cat #, E12055; Thermo Fisher Scientific, Inc.) to detect MMP activity in the aneurysmal aorta according to a previously described method (14). Briefly, freshly prepared aorta cryosections (10 µm) were washed with PBS twice and then incubated with a fluorogenic gelatin substrate in a dark humidified chamber (2 h, 37°C). The negative control sections were incubated with 1,10-phenanthroline (1 h, 37°C) before the addition of substrate. The MMP activity of a tissue sample was calculated by subtracting the fluorescence intensity of its corresponding negative control. The relative MMP activity of C57BL/6N mice was calculated by normalizing the fluorescence signal intensity in the aorta of C57BL/6J aneurysmal aortae, which was set as 1.

### Statistical analysis

The quantitative data are expressed as the mean ± standard deviation (SD). Student's *t* test was conducted for normally distributed data; otherwise, the nonparametric Mann‒Whitney test was used. Semiquantitative data, such as histological score data, are presented as medians and interquartile ranges, and the nonparametric Mann‒Whitney test was performed. Two-way ANOVA with multigroup comparisons was performed for aortic diameter comparisons (two timepoints). GraphPad Prism 9.0 was used for statistical analysis, and *P* < 0.05 was considered a statistically significant difference.

## Results

### Baseline levels of the C57BL/6N and C57BL/6J mouse substrains on body weight, blood pressure and abdominal aortic diameters

**Figure 1:**
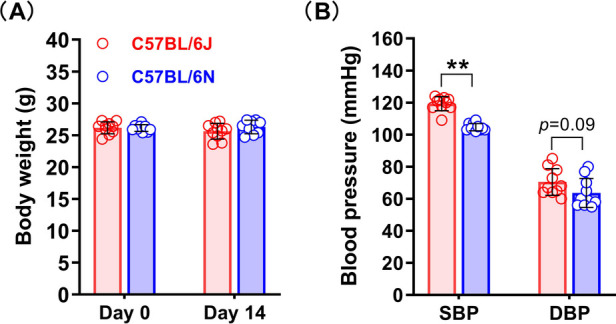
Body weight and blood pressure of the C57BL/6N and C57BL/6J substrains. **(A)** Body weights of the C57BL/6N and C57BL/6J substrains on days 0 and 14 after surgery (*n* = 9–10/group). **(B)** Blood pressure baseline levels of the C57BL/6N and C57BL/6J substrains. Student's *t* test was used to compare two groups. SBP, systolic blood pressure; DBP, diastolic blood pressure. **P < 0.01.

**Figure 2:**
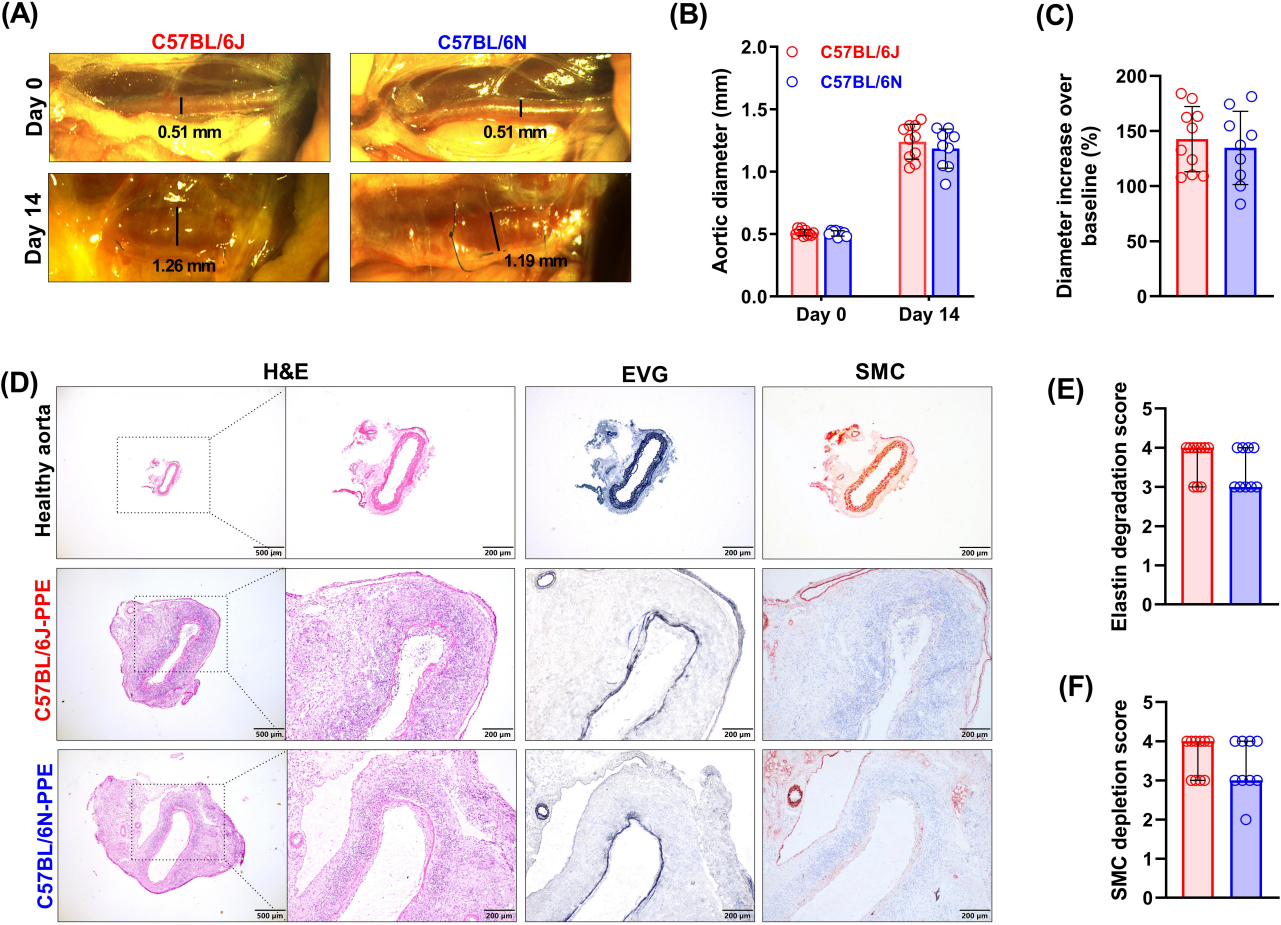
PPE infusion induced an increase in aortic diameter, medial elastin degradation and SMC depletion in C57BL/6J and C57BL/6N mice. **(A)** Representative images of abdominal aortas on days 0 and 14 after surgery (*n* = 9–10/group). **(B)** Aortic diameters on Day 0 (baseline) and Day 14 (sample collection timepoint) after PPE infusion. Two-way ANOVA with multigroup comparisons was performed. **(C)** Increase in aortic diameter compared with baseline (%). Student's *t* test was used to compare two groups. **(D)** Representative images of histopathological staining (H&E, EVG and SMC IHC staining) of healthy aorta or aneurysmal aortic segments in frozen sections. **(E**,**F)** Histological grading and semiquantification of aortic medial elastin and SMC depletion. A nonparametric Mann–Whitney test was performed.

To ensure that the baseline information of the two substrains of mice was similar before PPE infusion and to reduce experimental error, we examined the mouse body weights, blood pressures (two new separate mouse cohorts, age and body weight matched) and aortic diameters. As shown in Figure 1. The baseline data of the two substrains of mice were very similar in body weight. However, the systolic blood pressure of the C57BL/6N substrain was naturally lower than that of the C57BL/6J substrain in these two cohorts (Figure 1B). the baseline levels of abdominal aortic diameter were also similar between two sub strains (Figure 2B).

### PPE infusion successfully induces experimental AAAs in both C57BL/6N and C57BL/6J mouse substrains

To elucidate whether the C57BL/6N mouse substrain is as susceptible as the C57BL/6J mouse substrain to PPE-induced experimental AAA formation, all the mice were given transient luminal infusions of PPE. Fourteen days after PPE infusion, the diameters of the abdominal aortas were increased substantially in both mouse substrains, from an average of 0.51 to 1.24 mm in the C57BL/6J substrain and from 0.51 to 1.18 mm in the C57BL/6N substrain (Figures 2A,B). All the mice of both substrains presented an increase in diameter of more than 50% (143% and 135% increase in the C57BL/6J and C57BL/6N mice, respectively), meeting the criteria from AAA model establishment (Figure 2C). To measure changes in aortic diameters continuously by an ultrasound imaging, we performed new surgery for intraluminal PPE infusion in both C57BL/6J and C57BL/6N mice. As shown in Supplementary Figure S1, the difference in diameter between C57BL/6N and C57BL/6J mice after PPE infusion was not statistically significant. In the present study, the survival rate was 83% (10/12) in C57BL/6J mice and 75% (9/12) in C57BL/6N mice. AAA rupture is very rare in PPE infusion-induced mouse AAA models. No ruptures occurred in the current study. In both substrains of mice, AAAs were successfully induced by PPE infusion, and the difference between the two substrains was not significant in terms of diameter increase.

### PPE infusion induces medial elastin destruction and SMC depletion in both substrains

Medial SMCs and elastin are the main structural components of the aorta that maintain normal vascular function. Histological analysis revealed that in two mouse substrains, PPE infusion induced local aortic wall inflammation, elastin lysis or breakage, and SMC depletion (Figure 2D). In controlled healthy abdominal aorta, a clear vascular wall structure, neatly arranged SMCs, and continuous elastin layers can be seen (Figure 2D). The histological score for aortic elastin degradation was 4 (3–4, median with interquartile range) in C57BL/6J mice and 3 (2–3) in C57BL/6N mice (Figures 2D,E). According to the results of the SMC IHC staining analysis, the medial SMC loss scores of the two mouse strains were similar to the elastin scores (Figures 2D,F). PPE infusion induced medial elastin destruction and SMC depletion in both substrains, but the differences were not significant.

### Leukocyte subsets accumulate in aortic aneurysmal lesions

**Figure 3:**
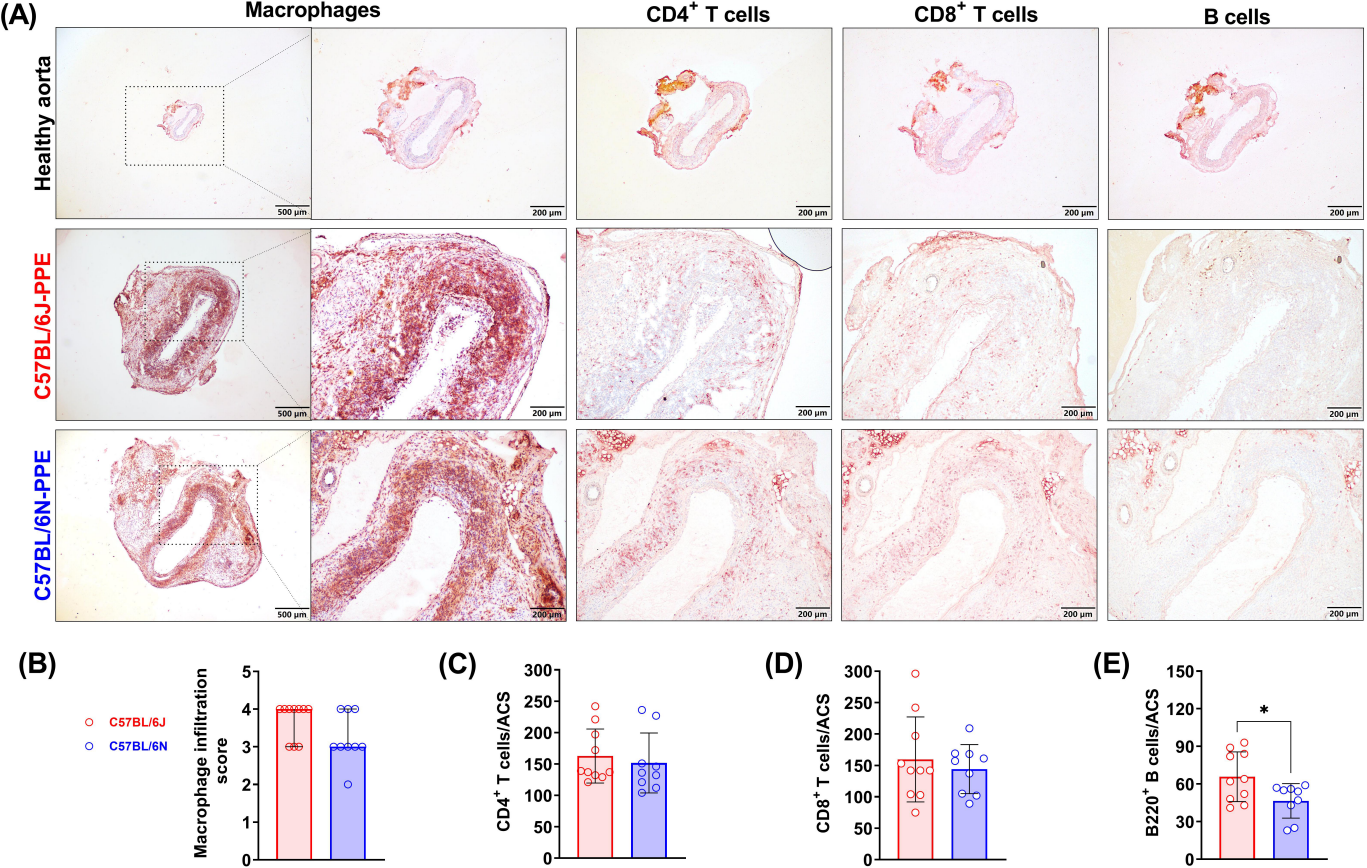
PPE infusion induced aortic leukocyte infiltration in experimental AAAs from C57BL/6J and C57BL/6N mice. **(A)** Representative images of aortic infiltrated macrophages, *T* cells and B cells. **(B**–**E)** Quantification or semiquantification of numbers of leukocyte subsets per ACS (*n* = 9–10/group). For data that were normally distributed, Student's *t* test was conducted (T cells and B cells), and the nonparametric Mann–Whitney test was used for semiquantification of the macrophage score. *P < 0.05.

Abnormal leukocyte infiltration is one of the most important promoters of AAA formation and progression. After PPE infusion, many macrophages infiltrated the aneurysmal lesions, and the macrophage infiltration scores were similar in the two mouse substrains, with no significant differences [graded as 4 (3–4) in C57BL/6J mice and 3 (3–4) in C57BL/6N mice] (Figures 3A,B). Similar to the macrophage results, PPE infusion also induced CD4^+^ *T* cell (163/ACS in C57BL/6J mice and 152/ACS in C57BL/6N mice) and CD8^+^ *T* cell infiltration (160/ACS in C57BL/6J mice and 144/ACS in C57BL/6N mice) (Figure 3C,D). However, PPE infusion recruited more B cells to the aneurysmal lesion site in C57BL/6J mice than in C57BL/6N mice (66/ACS vs. 47/ACS, respectively, *P* < 0.05) (Figure 3E).

### MMP2 and MMP9 expression and activity tests in aneurysmal lesions

**Figure 4:**
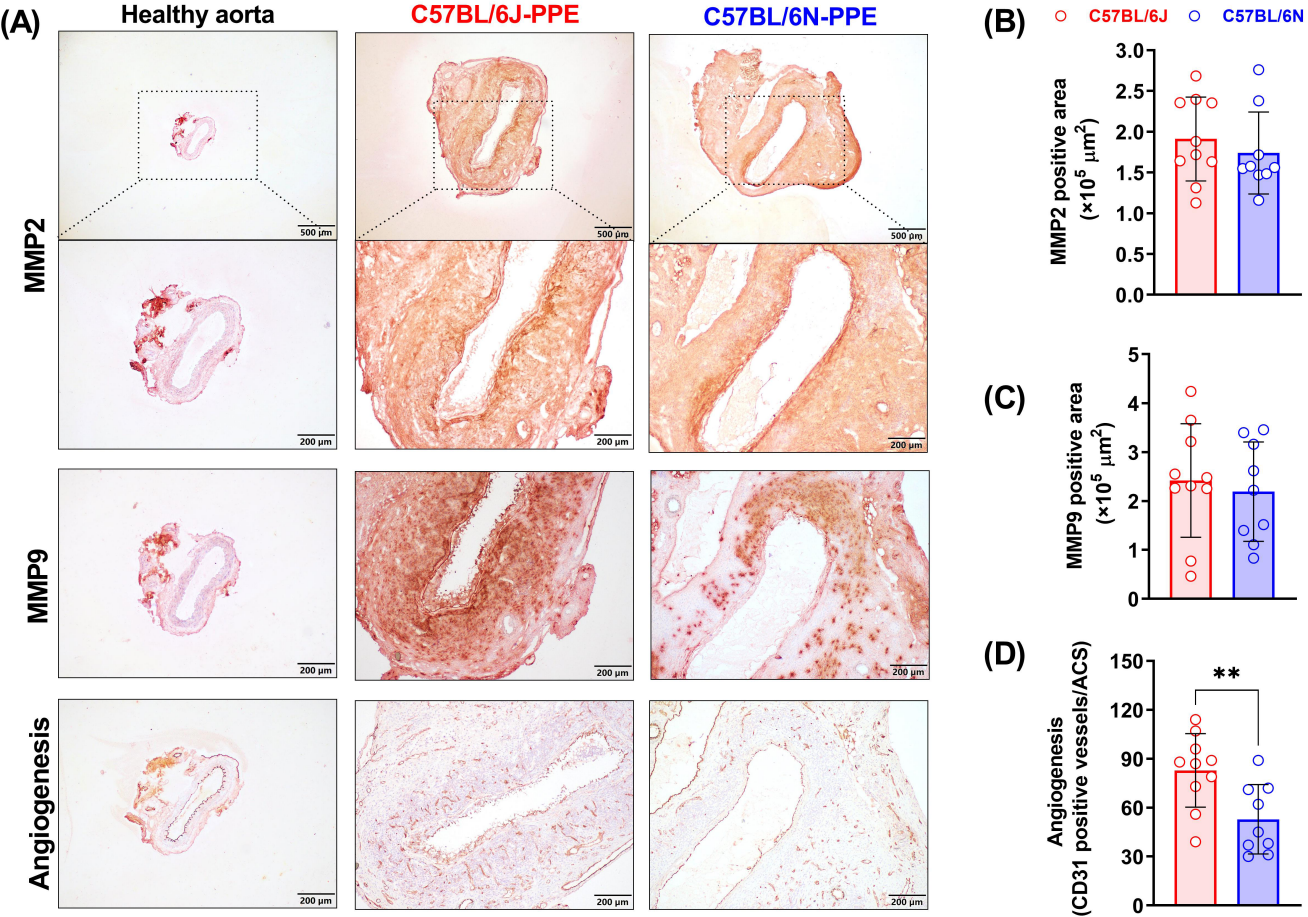
Aortic MMP (2 and 9) expression and mural angiogenesis after PPE infusion in C57BL/6J and C57BL/6N mice. **(A)** Representative IHC images of MMP2, MMP9 and mural neovessels in healthy aorta or aneurysmal aortas. **(B**–**D)** Quantification of MMP2 and MMP9 positive areas and mural neovessel numbers in the aneurysmal aorta. Student's *t* test was conducted (*n* = 9–10/group). **P < 0.01.

MMPs, especially MMP2 and MMP9, play important roles in the pathogenesis of AAA. PPE infusion increased the expression levels of MMP2 and MMP9 in aortic aneurysmal lesions (Figure 4A). However, when the areas that stained positive for MMP2 and MMP9 were compared between C57BL/6J mice and C57BL/6N mice, there was no significant difference (Figures 4A–C). MMPs produced by aortic-infiltrated macrophages and other cells may contribute to elastin and extracellular matrix destruction. The *in situ* enzymatic activities of MMPs also suggested that PPE infusion-induced MMP2 and MMP9 production may accelerate medial elastin degradation in both mouse substrains (Supplementary Figure S2).

### PPE infusion induces more neovessel sprouting in C57BL/6J mice

Abnormal mural angiogenesis is a pro-aneurysmal factor that represents another pathological feature of PPE-induced AAA development. CD31 staining revealed no or few neovessels in nonaneurysmal aortae. Although PPE infusion promoted mural neovessel sprouting in both mouse substrains, more neovessels were observed in C57BL/6J mice. On day 14 after PPE infusion, the mural neovessel number was 83/ACS in C57BL/6J mice, which was significantly greater than that in C57BL/6N mice (53/ACS) (Figure 4D).

## Discussion

Modeling human AAAs in animal models to study their pathogenesis, potential therapeutic targets, and drug safety has gradually become the mainstream research paradigm in this field (10). In AAA-related studies, the PPE-induced rodent aneurysm model, especially the mouse model, is still one of the most widely used models (10–13). It has been reported that the susceptibility to AAA formation after PPE infusion is associated, at least in part, with different strains (5, 8, 15). Substrain differences in the same strain, such as C57BL/6J and C57BL/6N, have also been reported to result in unequal susceptibility to the same cardiovascular disease modeling method (9, 16). Although a previous study revealed that C57BL/6J mice are more sensitive to Ang II-induced dissected aneurysms than are C57BL/6N mice (9), these two substrain mice did not significantly differ in their responses to PPE challenge in this study.

In general, a hyperlipidemic background (ApoE or LDLR deficiency) is needed to determine the chronic inflammation status in an Ang II-induced dissected aneurysm mouse model. Owing to the mutation of the nicotinamide nucleotide transhydrogenase (Nnt) exons 7–11, the Nnt protein is naturally deleted in C57BL/6J mice. Therefore, compared with C57BL/6N VSMCs, C57BL/6J VSMCs display increased oxidative stress, oxidative DNA damage and a stronger inflammatory phenotype (7, 17). Compared with C57BL/6N mice, C57BL/6J mice generally have greater systolic blood pressure, and this difference is exacerbated by angiotensin II infusion (18). These findings may explain why C57BL/6J mice are more susceptible to Ang II induction. However, in a PPE-induced mouse experimental AAA model, mechanical tension induced by pressure infusion and subsequent local inflammation caused by PPE penetrating into the aortic wall promoted aneurysm formation (10). Although this study revealed that the abdominal aortic diameter of C57BL/6N mice was slightly smaller than that of C57BL/6J mice after PPE infusion, the difference was not significant and did not affect the feasibility of the use of this substrain as an AAA research tool.

PPE infusion causes acute local stimulation to the aortic wall, which is different from Ang II-induced chronic systemic challenge. Nnt deficiency has been shown to cause minor functional changes in SMC function in C57BL/6J mice in previous studies (9, 18, 19). Therefore, Nnt deficiency may not be sufficient to affect PPE infusion-induced aneurysm formation in C57BL/6N mice. Our findings are similar to those of previous reports that some mouse strains without Nnt deficiency, such as FVB/N and BALB/c, are also susceptible to elastase-induced abdominal aortic aneurysm formation (15). Notably, several lesion indicators, such as the number of mural neovessels and infiltrating B cells, were greater in C57BL/6J mice after PPE infusion than in C57BL/6N mice. Aortic wall neovascularization is a characteristic pathological features of human AAA disease and considered to play critical roles in the development and rupture of AAA (20–24). Previous study found that invading neovessels are relevant sources of MMPs in the AAA wall and may substantially contribute to aneurysm wall instability (25). The HIF/ROS/VEGF pathway was found to be a master regulator of angiogenesis (26, 27). Nnt deficiency in the C57BL/6J substrain leads to the generation of more ROS than in the C57BL/6N substrain (28, 29). Oxidative stress can activate a variety of transcription factors, including NF-κB, Nrf2 and HIF-1α, and may promote angiogenesis (30, 31). A previous study on the effects of Nnt in perinatal hypoxia on the cerebral vasculature also revealed more feathery vessels in the C57BL/6J substrain than in the C57BL/6N substrain (28). As a central regulator of mitochondrial ROS levels, Nnt could have a significant effect on inflammation and ROS-driven cardiovascular disease. Deficiency of Nnt usually results in a stronger inflammatory response, which may explain why more B cells were observed in the C57BL/6J substrain (32). However, the detailed molecular mechanism is still unclear. In the PPE-induced AAA model, elastase infusion injury initially induces acute inflammation before day 7 and then transitions to a chronic inflammatory response involving the recruitment of monocytes/macrophages and T cells from day 10 to day 14. Later events involve adaptive immune responses and connective tissue repair (15). The deficiency of Nnt in the C57BL/6J substrain allows it to maintain a “chorionic inflammation” status and may lead to the earlier recruitment of B cells than in the C57BL/6N substrain. A long-term follow-up study may be needed to clarify this hypothesis in the future. Overall, the histological lesion phenotype of aneurysms in C57BL/6N mice was also typical. The C57BL/6N mouse substrain is viable to PPE infusion and can be used for AAA research.

In this study, we demonstrated that the C57BL/6N mouse strain, a widely used mouse strain, is a viable option for establishing a model of elastase-induced AAA development. This finding could dispel confusion when researchers want to study AAAs with a C57BL/6N genetic background in wild-type or genetically modified mice. Notably, the current study was limited in that only a PPE-induced AAA model was established. Whether C57BL/6N mice are also viable options for other methods of model establishment, such as the perivascular application of CaCl_2_, remains to be confirmed. Another limitation is the continuous changes of the aortic diameters can not be obtained by directly measurement method. As a supplement, we performed new surgery for intraluminal PPE infusion in both mouse substrains and measured the continuously changes of aortic diameters by an ultrasound imaging (Supplementary Figure S1). The third limitation of the current study is only male mice were chosen in AAA modeling. It is recognized that biological sex is critical in experimental AAA models, and whether female C57BL/6N mice are also suitable for PPE induced AAA modeling remains to be confirmed in future.

## Data Availability

The raw data supporting the conclusions of this article will be made available by the authors, without undue reservation.

## Supplementary material

Supplementary Figure S1 Measurement the changes of the aortic diameters after PPE infusion. **(A)** Representative ultrasound images for infrarenal aortic diameters at baseline (day 0), 7- and 14-days following PPE infusion. **(B)** Infrarenal aortic diameter before (day 0, baseline), and 7, and 14 days following PPE infusion. *n* = 8–10 mice per group.

Supplementary Figure S2 MMP activity analysis in PPE induced aneurysmal aortic segments. **(A)** C57BL/6J and C57BL/6N mice were given transient luminal infusions of PPE MMP activities of aneurysmal aortic tissues were determined by *in situ* zymography. **(B)** Fold change was calculated relative to the C57BL/6J mice; *n* = 9–10 mice per group.
