# Supplementary figures and images for: The C57BL/6N mouse substrain is a viable model of elastase-induced abdominal aortic aneurysm
Source: Front Cardiovasc Med. 2024 Sep 27;11:1462032. doi: 10.3389/fcvm.2024.1462032 (PMC11466807; doi:10.3389/fcvm.2024.1462032)

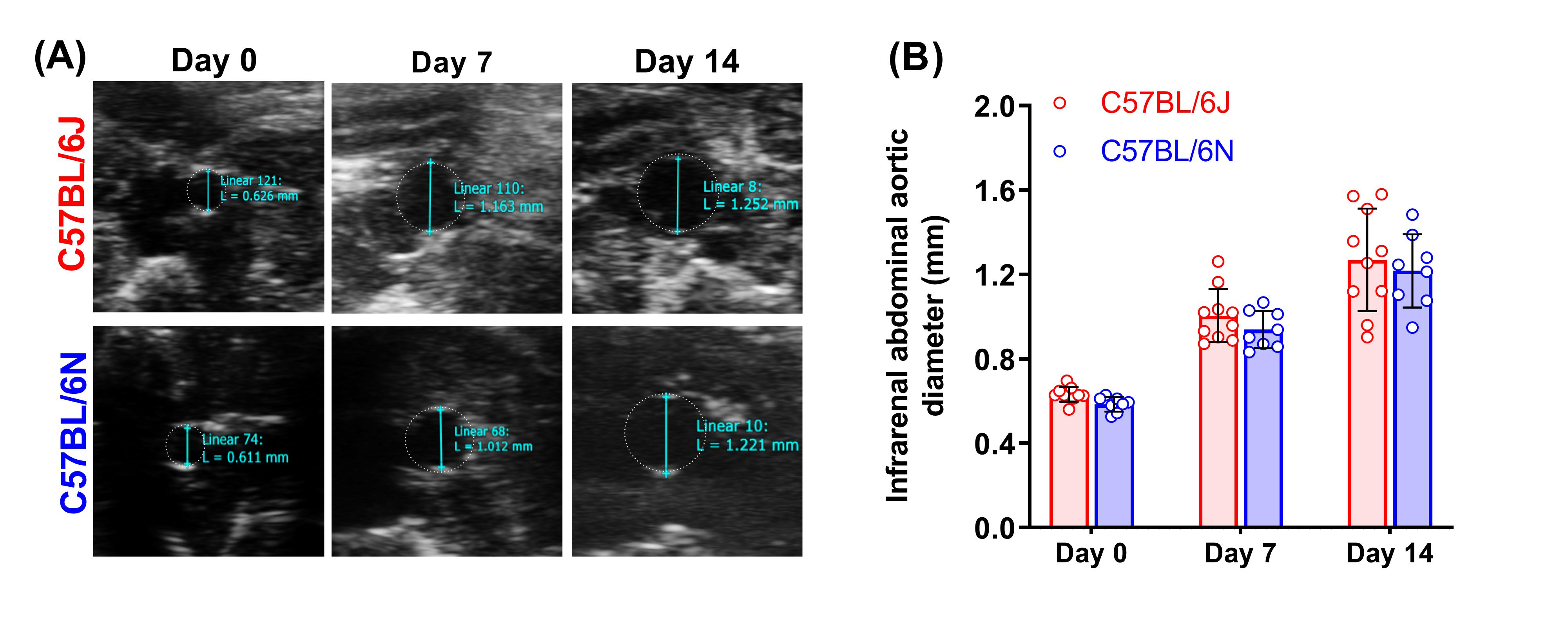

Supplement: Supplementary Figure S1 — Measurement the changes of the aortic diameters after PPE infusion. (A) Representative ultrasound images for infrarenal aortic diameters at baseline (day 0), 7- and 14-days following PPE infusion. (B) Infrarenal aortic diameter before (day 0, baseline), and 7, and 14 days following PPE infusion. n = 8–10 mice per group. [file Image1.jpeg]

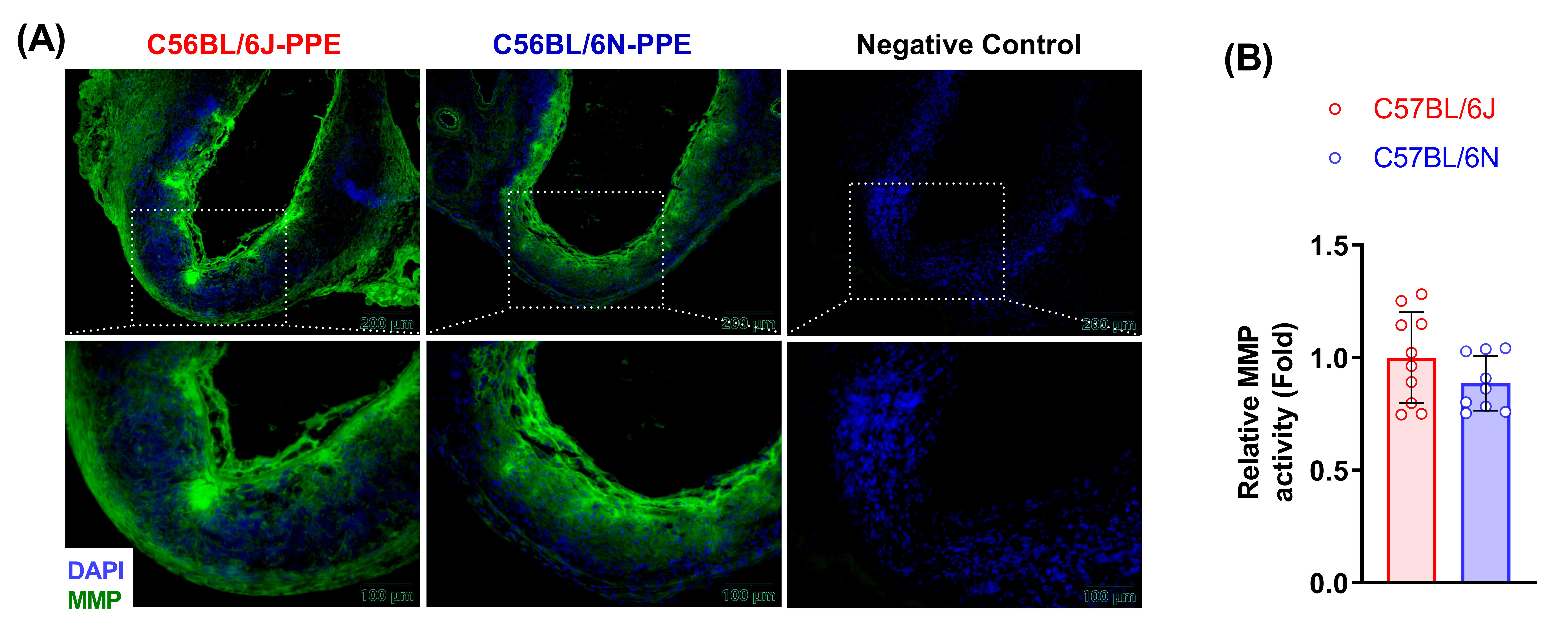

Supplement: Supplementary Figure S2 — MMP activity analysis in PPE induced aneurysmal aortic segments. (A) C57BL/6J and C57BL/6N mice were given transient luminal infusions of PPE MMP activities of aneurysmal aortic tissues were determined by in situ zymography. (B) Fold change was calculated relative to the C57BL/6J mice; n = 9–10 mice per group. [file Image2.jpeg]
