# Supplementary material for: The C57BL/6N mouse substrain is a viable model of elastase-induced abdominal aortic aneurysm
Source: Front Cardiovasc Med. 2024 Sep 27;11:1462032. doi: 10.3389/fcvm.2024.1462032 (PMC11466807; doi:10.3389/fcvm.2024.1462032)
Supplement: Supplementary file 3 [file Table1.docx]

**Supplementary table 1. Antibodies and other major reagents**

| Antibodies | | | | |
| --- | --- | --- | --- | --- |
| Target antigen | Catalog # | Dilution ratio | Host | Source |
| Mouse SMC alpha actin | 19245S | 1:200 | rabbit | Cell Signaling Technology |
| CD68 | 137002 | 1:200 | rat | Biolegend |
| CD4 | 100402 | 1:200 | rat | Biolegend |
| CD8 | 100702 | 1:200 | rat | Biolegend |
| CD45R | 103202 | 1:200 | rat | Biolegend |
| CD31 | 102402 | 1:200 | rat | Biolegend |
| MMP2 | AF1488 | 1:200 | Goat | R&D Systems |
| MMP9 | AF909 | 1:200 | Goat | R&D Systems |
| anti-rat antibody | BA-9400 | 1:400 | goat | Vector Laboratories |
| anti-goat IgG | 705-065-003 | 1:400 | donkey | Jackson Immuno Research |
| anti-goat IgG | BA-1000-1.5 | 1:400 | goat | Vector Laboratories |
| Additional reagents | | | | |
| Name | Catalog # | Dilution ratio | Host | Source |
| Elastase | E-1250 | 1:20 | Porcine pancreas | Sigma-Aldrich Corp |
| streptavidin-peroxidase conjugate | 016-030-084 | 1:400 | NA | Jackson Immuno Research |
| AEC substrate kit | SK-4200 | NA | NA | Vector Laboratories |
